# Development and consensus process for a clinical pathway for the assessment and management of chemotherapy-induced peripheral neuropathy

**DOI:** 10.1007/s00520-022-07024-3

**Published:** 2022-04-08

**Authors:** David Mizrahi, David Goldstein, Matthew C. Kiernan, Louisa Robinson, Omali Pitiyarachchi, Susan McCullough, Phil Mendoza-Jones, Peter Grimison, Frances Boyle, Susanna B. Park

**Affiliations:** 1 The Daffodil Centre, The University of Sydney, a joint venture with Cancer Council NSW, Sydney, Australia; 2 Faculty of Medicine and Health, Prince of Wales Clinical School, UNSW Sydney, Sydney, Australia; 3 Department of Medical Oncology, Prince of Wales Hospital, Sydney, Australia; 4 Brain and Mind Centre, The University of Sydney, Sydney, Australia; 5 Department of Neurology, Royal Prince Alfred Hospital, Sydney, Australia; 6 Translational Cancer Research Network Consumer Advisory Panel, Sydney, Australia; 7 Chris O’Brien Lifehouse, Sydney, Australia; 8 Sydney Medical School, Faculty of Medicine and Health, University of Sydney, Sydney, Australia; 9 Patricia Ritchie Centre for Cancer Care and Research, Mater Hospital, North Sydney, Australia

**Keywords:** Cancer, Oncology, Clinical pathway, Assessment, Peripheral neuropathy, CIPN

## Abstract

**Background:**

Cancer patients treated with neurotoxic chemotherapy are at risk of developing neurological symptoms that can impact functional capacity and quality of life. However, there are no standardised pathways to assess and manage chemotherapy-induced peripheral neurotoxicity (CIPN). This study aimed to determine consensus on statements regarding a CIPN assessment and management clinical pathway.

**Methods:**

A CIPN clinical pathway (CIPN-path) was developed and reviewed by an expert multi-disciplinary panel and consumers. Agreement with 18 statements regarding four content themes (pretreatment review, screening and assessment, management and referral, and CIPN-path feasibility) were assessed by 70 Australian respondents (68 health professionals, 2 consumers), using a 2-stage Delphi survey process to reach consensus. Respondents rated statements using a 5-point Likert scale to determine the level of agreement, with consensus defined as ≥ 80% of respondents agreeing with each statement.

**Results:**

The consensus was reached for 14 of 18 items after stage 1 and all items after stage 2. Feedback was obtained for all items to refine the CIPN-path. There was an agreement on important characteristics of the CIPN-path, including pretreatment screening, regular patient-reported assessment, and a stepped-care approach to investigating and managing symptom burden. There was a lack of agreement on who should oversee CIPN assessment, which may differ according to the structure and resources of each site.

**Conclusions:**

There was an overall agreement concerning the CIPN-path to assess and manage CIPN, which may be adapted accordingly to the resources of each clinic. The CIPN-path may assist teams across different health services in identifying CIPN symptoms, aiding decision-making, and reducing morbidity from CIPN.

**Supplementary Information:**

The online version contains supplementary material available at 10.1007/s00520-022-07024-3.

## Introduction

Chemotherapy-induced peripheral neuropathy (CIPN) is a major neurological side effect of the treatment of cancer, associated with early cessation of treatment and long-lasting disability [1, 2]. There is now a burgeoning population of cancer survivors [3, 4] potentially at risk of CIPN developing during and following treatment with neurotoxic platinum compounds, taxanes, vinca alkaloids, thalidomide, and bortezomib [1]. CIPN commonly induces paraesthesia, pain, and numbness in the hands and feet, producing sensory ataxia and significantly heightened fall risk [5]. Despite successful treatment, 77% of surveyed Australian cancer survivors treated with neurotoxic chemotherapies reported persistent neuropathy [6], with up to 40% of cancer survivors left with long-term functional disability and reduced quality of life due to CIPN [7]. The lack of proven preventative strategies and sensitive assessment tools has hampered the development of clinical trials and the optimal delivery of health services in this area.

A suite of patient-centred CIPN assessment tools have been developed, yet these remain under-utilised in clinical practice [8]. The most common CIPN assessment tool is the clinician-administered National Cancer Institute Common Terminology for Adverse Events (neuropathy sensory subscale), which lacks inter-observer reliability [9] and may under-report toxicity compared to patient reports [10]. Although the deficits in neuropathy grading in clinical practice have long been recognised, and numerous patient-reported outcome measures have been developed for clinical use, this has not translated to widespread improvements in neuropathy assessment in routine oncology practice.

A key barrier to the uptake of improved CIPN assessment is the lack of guidance available to identify optimal clinical screening procedures suitable for use across health delivery services. The best practice timing, frequency, and responsibility for CIPN screening are not well established. Capturing CIPN symptoms early or identifying those at increased risk may allow opportunities for closer neurological surveillance by treating oncology teams [11]. Additionally, with known functional deficits resulting from CIPN symptoms, including reduced mobility and increased falls risk [12, 13], there is an important clinical need to ensure patients with functional impairments receive adequate rehabilitation to preserve their quality of life.

Clinical pathways and clinical decision-making tools have been shown to improve health care process measures across diverse settings [14], reducing clinical variation and potentially improving outcomes [15, 16]. There are limited pathways outlining best practices for the assessment and management of CIPN across healthcare teams, with current guidelines synthesising evidence for preventative and treatment interventions [17, 18], without specifying implementation pathways. Limited data exist regarding CIPN assessment and management utilising multi-disciplinary models of care [19–21]. An important next step is to operationalise how guidelines can be translated for use into a health service delivery pathway to ensure best practice CIPN assessment and management. Thus, the development of a CIPN clinical practice pathway provides a framework to aid clinical decision-making across healthcare teams that care for cancer patients. Accordingly, the aim of this study was to develop a clinical pathway for the assessment and management of CIPN via a Delphi consensus process and obtain feedback from relevant stakeholders regarding the pathway and future implementation strategies.

## Methods

### Participants

The survey aimed to examine the views of relevant stakeholders in CIPN assessment and management in routine clinical practice in Australia. Recruitment of targeted health professionals, including medical oncologists, neurologists, nurses, and allied health professionals, as well as specialised CIPN researchers working in Australia, who have experience working with patients with cancer or could be referred patients with CIPN. Potential participants were identified through an established national CIPN research network known to the lead researchers, as well as identifying multi-disciplinary health professionals around Australia by searching the websites of cancer centres, who were invited to the study by email with a website link to the survey. Using the peer esteem snowballing method [22], participants were also invited to share the study invitation within their networks. The study was approved by the University of New South Wales Human Research Ethics Committee (HC210167).

### Clinical pathway development

The ‘CIPN clinical practice pathway for the assessment and management of chemotherapy-induced peripheral neuropathy’ (CIPN-path, Supplementary Material 1, Fig. 1) was developed to provide a health service delivery pathway for CIPN assessment, management, and decision-making. The CIPN-path development process included a 10-member expert advisory committee of medical oncologists (3), neurologists/neurophysiologists (2), nurses (2), allied health professionals (1), and patient consumers (2), which was created from an existing working party formed to examine CIPN assessment tools [8]. This initial group was selected to incorporate multidisciplinary expertise relevant to CIPN clinical practice and assessment. All members of the advisory committee were familiar with CIPN, although care was taken to ensure that varying levels of CIPN expertise were represented.

**Fig. 1:**
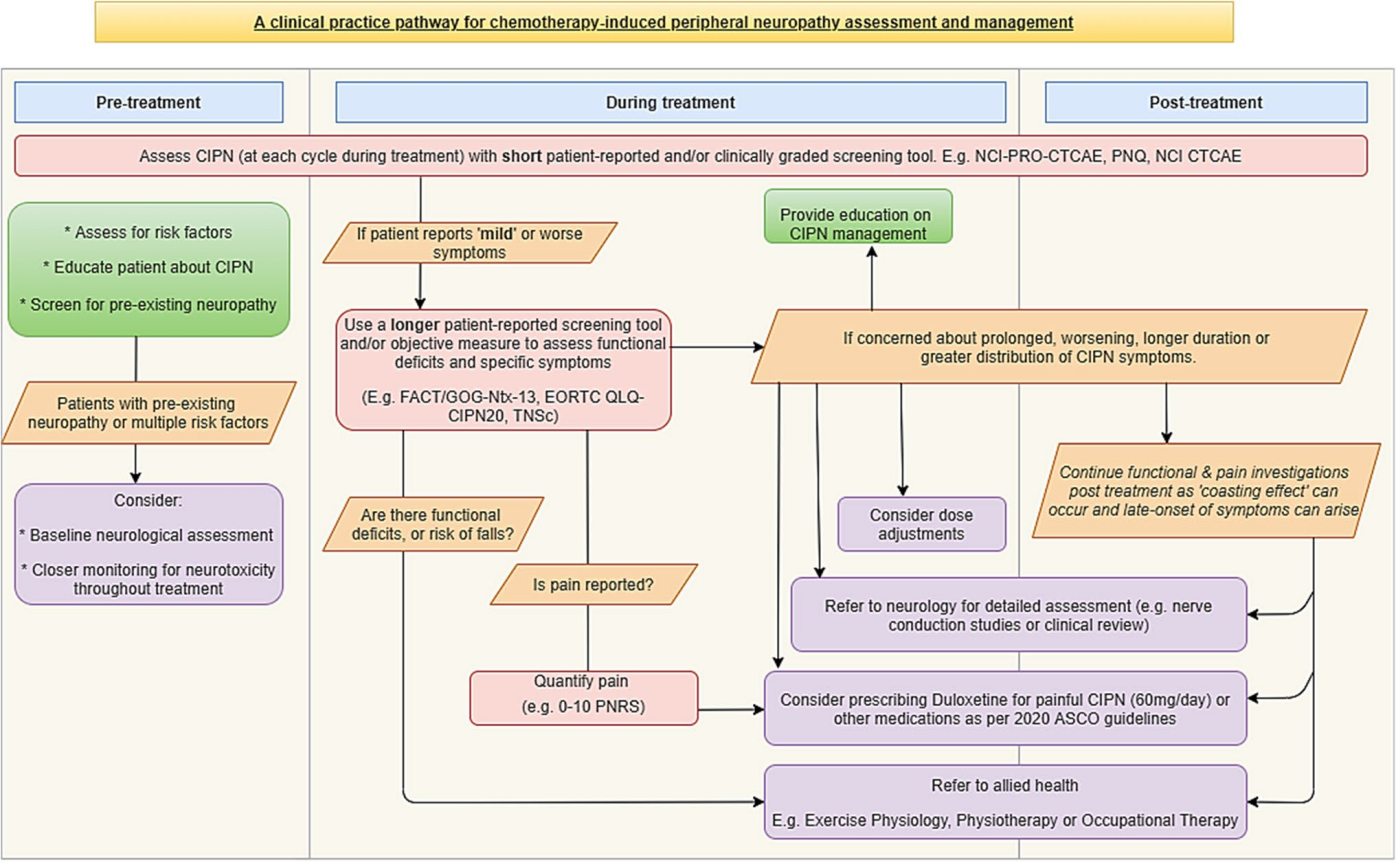
Flow chart of the clinical pathway for the assessment and management of CIPN

The CIPN-path translated evidence from existing literature and evidence-based guidelines on CIPN prevention and treatment, including the American Society of Clinical Oncology (ASCO) [17] and European Society of Medical Oncology guidelines (ESMO) [18]. This evidence was synthesised by research focused nurse with input from the first author before being reviewed by senior authors. Following this initial drafting, the draft clinical pathway was presented at relevant meetings to gain feedback. The pathway was electronically sent to advisory group members for comment, which comprised text and discussion-based comments. Advisory group members were given the opportunity to comment on the final draft pathway prior to distribution in the Delphi survey. The aim of the CIPN-path was to provide practical advice on how to operationalise current CIPN management evidence and guidelines into a health service delivery pathway. The CIPN-path spans three phases of chemotherapy treatment (pretreatment, during treatment, and posttreatment), in which four important themes were identified, including (1) screening and assessment, (2) management and treatment, (3) referral and supportive care, and (4) feasibility of the clinical pathway. To gauge consensus and feasibility for future implementation work, 18 statements regarding the content of the CIPN-path were developed by multidisciplinary members of the research team.

### Consensus generation: modified Delphi survey process

**Fig. 2:**
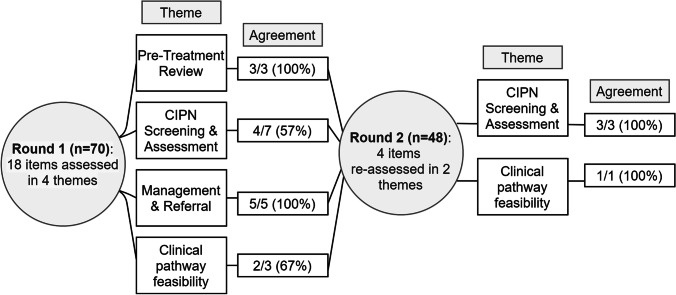
Agreement with items across four themes related to the CIPN clinical pathway in two stages in the Delphi survey process

A modified Delphi survey method was utilized [23], consisting of an iterative process to achieve consensus among a panel of experts. The survey was a two-stage process to reach consensus among the eighteen statements about the CIPN-path, with three open-ended questions, including which health professional(s) should be responsible for CIPN assessment, any suggested changes to the CIPN-path, and any other comments about the CIPN-path (Fig. 2). Participants completed the first stage (15–20 min) online via the email invitation weblink (REDCap V10.0.1). Six weeks later, participants were invited for the second stage, which included statements that did not reach consensus in the first stage (5–10 min). Open-field responses were included next to each statement that required consensus in the second stage so participants could provide feedback to enhance or justify any exclusions from the CIPN-path. Potential participants received reminders about the study 1 week after the original invitation for both stages. In both stages, participants indicated their agreement with all statements using a 5-point Likert scale from ‘strongly agree’ to ‘strongly disagree’. The consensus was defined as achieving ≥ 80% of respondents agreeing or strongly agreeing with each statement [24].

### Data analysis

Descriptive statistics were used to calculate the characteristics of the recruited population and the proportion of participants agreeing with each statement. Data were analysed using SPSS Statistics Version 27 (IBM, Armonk, NY, USA). We used content analyses via open text comments for individual items in the second stage to further explore participant responses. A mixed-methods approach was utilised to incorporate qualitative participant responses alongside quantitative findings.

## Results

### Participant characteristics

A total of 70 participants completed the first stage of the Delphi survey (out of 260 invited participants), and 48 completed the second stage. The recruited participants included 68 health professionals from multiple disciplines, including medical oncologists/haematologists (31%), nurses (24%), neurologists (16%), allied health professionals (11%; Table 1), and two consumers. Most participants were employed in a tertiary referral centre (63%) and were from an urban setting (89%). Employment experience was spread evenly across the cohort.

**Table 1:** Demographic characteristics of study sample

		*n*	Percentage
Gender	Female	45	64%
	Male	25	36%
Discipline	Medical oncologist/haematologist	22	31%
	Nurse	17	24%
	Neurologist	11	16%
	Allied health	8	11%
	Researcher	6	9%
	Other (e.g., neurophysiologist, rehabilitation physician)	4	6%
	Consumer	2	3%
Work setting	Tertiary referral centre	44	63%
	District/local hospital	12	17%
	Nonhospital based	8	11%
	Other	6	9%
Years’ experience^^^	1–5	11	16%
	6–10	20	29%
	11–20	18	26%
	20 +	19	28%
Rurality	Urban	62	89%
	Rural/regional	8	11%

^^^*N* = 68 as data is not included for two consumer participants

### Delphi consensus process

Following the first stage of the Delphi survey, 4 of the 18 items across 4 themes did not reach a consensus. These items were related to CIPN screening and assessment (theme 1) and the feasibility of implementing the CIPN-path (theme 4) and are discussed further below. In the second stage, these four items were resurveyed with open-text feedback and general feedback on the entire clinical pathway collected. Levels of agreement for all individual items are presented in Table 2.

**Table 2:** Consensus achieved for all items related to the CIPN clinical pathway in both stage 1 and 2

	Stage 1 (%)			Stage 2 (%)		
*Themes and individual items*	Agree	Unsure	Disagree	Agreed	Unsure	Disagree
***Pretreatment review***						
Patients should receive education about CIPN prior to treatment	100					
Medical history should be obtained to identify potential risk factors for CIPN	98.5	1.5				
Patients with preexisting peripheral neuropathy or who are at high risk should be considered for pretreatment neurological assessment or closer monitoring	94.2	2.9	2.9			
***CIPN screening and assessment***						
CIPN screening and assessment should continue to occur at follow-up visits after treatment has finished	97.0	1.5	1.5	-		
Screening for CIPN should occur at every treatment cycle	94.2	2.9	2.9	-		
Patients who are flagged with CIPN should be followed up with longer assessment tools to assess symptoms and impact on daily function	94.2	5.8	-	-		
Responses from patient-reported screening tools allow the clinical team to grade the patient’s CIPN	84.1	10.1	5.8	-		
The screening and assessment options recommended in the clinical pathway are appropriate	75.7	21.3	3.0	97.9	2.1	-
A short patient-reported questionnaire is the most appropriate tool for use in initial CIPN screening	72.5	23.2	4.3	95.8	-	4.2
Specific staff member(s) should be clearly designated as responsible for CIPN screening	52.9	29.5	17.6	81.3	4.1	14.6
***Management and referral***						
Specific CIPN symptoms, including neuropathic pain, balance impairment and falls risk, and sleep disturbances, may warrant closer monitoring, additional investigation, or referral to other care providers	98.5	1.5	-	-		
All patients should be encouraged to be physically active during treatment to minimise impairments to physical function	98.5	1.5	-	-		
Patient education about CIPN symptom management is important and should consider safety measures to reduce the risk of falls and thermal injury as well as foot care	98.5	1.5	-	-		
If patients have functional deficits or risk of falls, referral to allied health such as an exercise physiologist/physiotherapist or occupational therapist will assist to improve functional capacity	97.0	3.0	-	-		
Patients with CIPN symptoms that are prolonged, worsening, longer duration, or increasing in distribution should be referred to a neurology service	86.4	12.1	1.5	-		
***CIPN clinical pathway feasibility***						
Implementing this CIPN clinical pathway may lead to improved CIPN outcomes in patients	85.9	12.5	1.6	-		
The proposed clinical pathway should be tailored to the specific resources available	83.3	13.7	3.0	-		
The proposed clinical pathway represents a realistic and achievable clinical process	72.7	21.2	6.1	89.5	10.5	-

Consensus is defined as agreeing or strongly agreeing with each statement. Statements with < 80% consensus were reassessed in a 2nd stage, which included opportunities for feedback

### Pretreatment review

The consensus was reached on all 3 items related to pretreatment review in the first stage (94–100% agreement). Participants agreed that a pretreatment review should incorporate patient education about CIPN, a medical history should be obtained to identify potential CIPN risk factors and patients with preexisting neuropathy, and those at high-risk for CIPN should be considered for pretreatment neurological assessment or closer monitoring of CIPN symptoms throughout treatment. One respondent suggested that “*patients at risk of CIPN such as extreme obesity or diabetes should be evaluated by neurology prior to neurotoxic chemotherapy*”.

### CIPN screening and assessment

The consensus was reached on 4/7 items (57%) in stage 1 (53–97% agreement), which was the lowest of all themes assessed. In stage 1, participants agreed that CIPN screening and assessment should occur at each treatment cycle and continue post-treatment at follow-up visits. They also agreed that patients who report CIPN symptoms should be followed-up to determine symptom severity and impact on daily function and that patient-reported CIPN symptoms assist clinicians in clinically grading neuropathy severity. Three items in this theme did not reach consensus in stage 1: (1) The screening and assessment recommendations in the pathway are appropriate, (2) a short screening tool is appropriate for initial CIPN screening, and (3) a specific staff member should be dedicated to CIPN screening. These items did reach a consensus in stage 2 (Table 2).

The lack of consensus on these points is related to differences in views regarding appropriate assessment tools. One respondent noted that “a short patient-reported questionnaire seems most appropriate for initial screening as both easy and feasible for use in the clinic”. Conversely, another participant suggested, “implementing screening tools into routine care and escalation pathways involving other departments requires institutional buy-in and sufficient resourcing”.

Participants reported which of the health professional(s) should be responsible for CIPN screening and assessment: oncology nursing staff only (32%), medical oncologists and nursing staff (30%), all members of the multi-disciplinary team (e.g., medical oncologists, nursing staff, neurologists, allied health, 21%), medical oncologists only (13%) or neurologists only (5%). One respondent suggested that “regardless of the staff member involved, it is crucial they have experience with CIPN screening”. Another respondent suggested that “there are too many toxicities to screen for, so screening for all toxicities should be shared among clinician and nursing staff”. Some respondents were concerned by the suggestion of having numerous staff members to screen for CIPN, suggesting “if too many staff were involved, it would be challenging to achieve consistency” and “having a dedicated staff member responsible will ensure standardised screening occurs”, while others disagreed, stating that “decentralising screening among trained staff will be more efficient at detecting symptoms earlier and reducing delay for comprehensive assessment”.

### Management and referral

The consensus was reached on all 5 items in stage 1 (86–98% agreement). Regarding CIPN management and referral, participants agreed that symptoms resulting from CIPN, including neuropathic pain, balance impairment, and sleep disturbances, may warrant closer monitoring. Participants agreed that symptoms resulting from CIPN require further investigation or referral to other care providers. Participants agreed that patients with functional deficits should be considered for referral to allied health professionals. Participants agreed that patients should be encouraged to be physically active and that education should be provided for safety measures, including thermal management, balance, and foot care. The consensus was also achieved concerning the consideration of referral of patients with worsening or prolonged CIPN symptoms to a neurology service, although one respondent highlighted that “the subtleties of small fibre neuropathy can occur before it becomes clinically apparent, making it challenging to only rely on patients self-reporting symptom changes to trigger the longer assessments”.

### Feasibility of the clinical pathway

The consensus was reached on 2/3 items (67%) in stage 1 (73–86% agreement). Participants agreed that implementing the CIPN-path would improve CIPN outcomes for patients and that the proposed pathway should be adapted to each site depending on the needs of the population and the availability of resources. One item did not reach consensus in stage 1, which was that CIPN-path represented a realistic and achievable process. This item did reach consensus in stage 2 (Table 2).

A common theme reported by respondents was potential difficulties with incorporating neurology services into CIPN management, including that “timely access to neurology and NCS is a barrier at some sites”, “routine neurology review may not be helpful, or be feasible, particularly in busy clinics”, and that in rural areas “patients do not have access to publicly available neurologists or specialised allied health professionals”. However, it was also suggested that “patients with a significant risk of developing CIPN should have pre- or early treatment baseline NCS, which can assist to guide treatment and improve the sensitivity of early CIPN diagnosis”.

There was agreement from respondents on the utility of the CIPN-path, including “this pathway which includes an evidence-based approach can support delivering best practice to patients…with many pathways lacking in pre- and posttreatment steps”, “having a clear pathway will help staff and patients be aware of CIPN and the ongoing problems associated with it” and that CIPN is often “poorly addressed from diagnosis, with patients feeling ill-informed with no clear management pathway”.

## Discussion

This study aimed to achieve consensus regarding a newly developed clinical pathway that has operationalised CIPN assessment and management advice from current evidence for multidisciplinary health professionals who care for cancer patients treated with neurotoxic chemotherapies. CIPN is significant toxicity of cancer treatment, and screening is needed to minimise symptom burden both during treatment and in cancer survivorship. Accordingly, we assessed the feasibility and clinical utility of a newly developed clinical pathway for screening, assessment, and management of CIPN. Our results highlighted support for enhanced CIPN management in clinical practice to assist teams across different health services to identify CIPN symptoms, aid decision-making, and reduce morbidity from CIPN, as well as identify focus areas for future implementation strategies.

Broadly, the consensus was achieved regarding key aspects of the CIPN-path from most respondents in this study. These included the importance of pretreatment review [25] and patient education, as well as the timing of CIPN assessments and clarity regarding optimal directions to follow to ensure appropriate and timely referral and relevant management. The proposed clinical pathway allows for both individual and institutional preferences and is not overly prescriptive to aid in uptake. The CIPN-path suggests the initial use of short patient-reported screening questionnaires, with stepped-care escalation to comprehensive questionnaires in symptomatic patients, to trigger the referral and management cascade on an as-needed basis. Centralising care around patient-reported symptoms allows for medical teams to respond in real time and has been shown to reduce hospitalizations and co-morbidities in cancer patients [26]. Utilising patient-reported outcomes also facilitates patient-clinician communication, which is crucial, particularly in the event where clinicians do not adequately discuss CIPN [27]. Incorporating patient feedback may identify persisting disability earlier, which is of crucial importance to those who develop functional impairments or become at risk for falls, allowing timely referral to interventions including exercise physiology, physiotherapy, or occupational therapy to improve functional capacity and reduce this burden [12].

Although we achieved consensus on all elements included in the clinical pathway, in the first round there were four items that failed to achieve consensus. These concerning assessment and screening procedures include the selection of appropriate tools and which personnel should be tasked with CIPN screening. Furthermore, the consensus was not reached on the overall feasibility of the pathway and the appropriateness of the pathway recommendations in the first round. The short screening questionnaires proposed in the CIPN-path have been found to provide adequate screening compared to longer instruments [28]. Short instruments (< 3 items) were also preferred in a survey of clinicians due to limited resources available in busy oncology clinics [8]. However, shorter instruments may be limited in terms of comprehensiveness and ability to identify specific concerns and thus may be less acceptable to patients [29]. There was also a discrepancy among respondents as to which health professional should lead CIPN screening, with mixed responses between nursing, medical oncology, and incorporated throughout the team. Realistically, screening approaches would differ by team structure and resource availability. Clinical pathways have been shown to have the highest likelihood of being successfully implemented when adapted for each setting, which may differ based on staff resourcing, access to services, and synergies with other multi-disciplinary health professionals and departments [30].

Achieving successful implementation of a clinical pathway and documenting improvements in clinical care remains a key focus which will require additional methods and implementation frameworks to evaluate the clinical utility across health services. A recent study examined the implementation of a CIPN clinician-decision support algorithm into clinical practice, although it did not increase rates of CIPN assessment and adherence to evidence-based management [19]. Barriers reported by clinicians included lack of time and finding the algorithm a burdensome process, whilst the implementation plan was reported to be sub-optimal [19]. Importantly, this highlights that implementation frameworks are necessary to identify barriers and seek feedback from key personnel before the successful implementation of improved CIPN assessment and management processes [31]. Utilising such frameworks, including the consolidated framework for implementation research [32] or expert recommendations for implementing change [33], can provide targeted approaches to key implementation barriers. In addition to institutional change, providing sufficient patient education and promoting a trusting relationship with full disclosure of CIPN symptoms has been shown to facilitate clear patient-doctor communication and is pivotal to symptom management [34]. Patient-reported outcomes have demonstrated a crucial component in identifying symptom severity and facilitating shared decision-making decisions in this context [35].

The CIPN-path supports CIPN assessment and management before, during, and after neurotoxic treatment, via a multi-disciplinary team, incorporating patient-reported outcome measures and ensuring sufficient flexibility to be adaptable to the needs of healthcare teams across institutions. The CIPN-path supports patient-decision-making, which is particularly important in patient-clinician discussions regarding the risk of worsening CIPN versus the benefit of continuing the same treatment intensity, including the effect on the quality of life [36]. Clinical pathways for diabetic peripheral neuropathy have also suggested that early identification of neuropathic symptoms, managing comorbidities, and utilising multi-disciplinary care are pivotal in minimising symptom burden and improving clinical outcomes, although are similarly impacted by timely access to specialised resources [37]. Similar issues were raised in our qualitative analysis regarding timely access to services such as neurology and referral to allied health professionals with experience seeing patients with CIPN. This can be problematic in both urban settings and rural areas. Since delivery of appropriate care after screening is the highest predictor of improvements in patient outcomes [38], there is a crucial need to include a multi-disciplinary approach. However, while referral to specialised clinical services, including nerve conduction studies, is a component of the CIPN-path, they are not recommended for all patients and must be triaged by priority to avoid overwhelming numbers of referrals. Furthermore, the only pharmacological treatment highlighted in the CIPN-path is duloxetine, which is moderately recommended to treat painful CIPN in the ASCO guidelines [17]. However, there is less evidence surrounding its efficacy in routine clinical practice [39], and routine duloxetine use has been shown to be low (< 1%) among patients who received neurotoxic chemotherapy regimens in comparison to other neuropathic pain medications [40].

The response rate in this study was low (70/260 respondents (27%) in round 1 and 48 respondents in round 2), which may result in non-response bias and represents a limitation of the study. Although this is a common phenomenon in web-based research [41], it should be considered in the interpretation of our results. Furthermore, because the sample was identified from known networks with expertise in CIPN, responses may be biased in comparison to professionals without such expertise. Therefore, clinical implementation efforts will be essential to demonstrate the feasibility and acceptability of the CIPN-path in routine clinical practice. Our study used free-text responses for participants to provide suggestions, however, our recruited participants did not have the opportunity for cross-disciplinary interactions, such as via a focus group, that may allow for broader interactions of the CIPN-path across multi-disciplinary teams. Furthermore, this study did not include patient decision support frameworks, which would provide an important avenue to support patients and clinicians to operationalise the pathway and its recommendations. Such frameworks have been developed [36] but require further study to implement and optimize. Strengths of this study include a recruited sample largely represented by health professionals working clinically and specialised in cancer care, and thus in a critical position to comment on clinical management, which was sufficient for a Delphi study [42].

## Conclusions

The consensus was achieved for an evidence-based clinical pathway for the assessment and management of CIPN. Regular CIPN screening can be conducted before, during, and after treatment, whilst symptomatic patients should complete thorough assessments to determine whether multi-disciplinary care or treatment modifications are required. While the present study surveyed health professionals across multiple disciplines, additional considerations may be required to tailor the CIPN-path for health services that lack accessible multi-disciplinary care, including those in rural areas. Future studies should assess the efficacy of the CIPN-path, which is an important step towards clinical implementation of best practice CIPN assessment and management strategies. Adhering to such pathways may improve the identification of CIPN, reduce symptom severity and subsequently guide appropriate referrals and treatment modification to preserve patient quality of life.

## Supplementary Information

Below is the link to the electronic supplementary material.

Supplementary file1 (PDF 858 kb)

## Data Availability

The datasets generated during and/or analysed during the current study are not publicly available due to conditions set out by the approving ethics committee but are available from the corresponding author on reasonable request.

## References

[1] Park SB, Goldstein D, Krishnan AV, Lin CS-Y, Friedlander ML, Cassidy J, … Kiernan MC (2013) Chemotherapy-induced peripheral neurotoxicity: a critical analysis*.* CA Cancer J Clin. 63(6): 419–437 10.3322/caac.21204 24590861

[2] Li T, Mizrahi D, Goldstein D, Kiernan MC and Park SB (2021) Chemotherapy and peripheral neuropathy. Neurol Sci 42(10):4109–4121 10.1007/s10072-021-05576-6 34436727

[3] Ferlay J, Soerjomataram I, Dikshit R, Eser S, Mathers C, Rebelo M, … Bray F (2015) Cancer incidence and mortality worldwide: sources, methods and major patterns in GLOBOCAN 2012*.* Int J Cancer. 136(5): E359–86 10.1002/ijc.29210 25220842

[4] AIHW, Australian Institute of Health and Welfare: cancer in Australia 2019. Cancer series no. 119. (2019)

[5] Kolb NA, Smith AG, Singleton JR, Beck SL, Stoddard GJ, Brown S, Mooney K (2016). The association of chemotherapy-induced peripheral neuropathy symptoms and the risk of falling. JAMA Neurol.

[6] Battaglini E, Goldstein D, Grimison P, McCullough S, Mendoza-Jones P, Park S (2021) Chemotherapy-induced peripheral neurotoxicity in cancer survivors: predictors of long-term patient outcomes. J Natl Compr Canc Netw 19(7):821–828 10.6004/jnccn.2021.7026 34340206

[7] Tan AC, McCrary JM, Park SB, Trinh T, Goldstein D (2019). Chemotherapy-induced peripheral neuropathy-patient-reported outcomes compared with NCI-CTCAE grade. Support Care Cancer.

[8] McCrary JM, Goldstein D, Boyle F, Cox K, Grimison P, Kiernan MC, … Park SB (2017) Optimal clinical assessment strategies for chemotherapy-induced peripheral neuropathy (CIPN): a systematic review and Delphi survey*.* Support Care Cancer. 25(11): 3485–3493 10.1007/s00520-017-3772-y 28589310

[9] Postma TJ, Heimans JJ, Muller MJ, Ossenkoppele GJ, Vermorken JB, Aaronson NK (1998). Pitfalls in grading severity of chemotherapy-induced peripheral neuropathy. Ann Oncol.

[10] Sasane M, Tencer T, French A, Maro T, and Beusterien KM (2010) Patient-reported outcomes in chemotherapy-induced peripheral neuropathy: a review. J Support Oncol 6(8):e15–e21

[11] Velasco R, Bruna J, Briani C, Argyriou AA, Cavaletti G, Alberti P, … Kalofonos HP (2014) Early predictors of oxaliplatin-induced cumulative neuropathy in colorectal cancer patients. J Neurol Neurosurg Psychiatr 85(4): 392–398 10.1136/jnnp-2013-305334 23813745

[12] Winters-Stone KM, Horak F, Jacobs PG, Trubowitz P, Dieckmann NF, Stoyles S, Faithfull S (2017). Falls, functioning, and disability among women with persistent symptoms of chemotherapy-induced peripheral neuropathy. J Clin Oncol.

[13] McCrary, Goldstein D, Trinh T, Timmins HC, Li T, Menant J, … Park SB (2019) Balance deficits and functional disability in cancer survivors exposed to neurotoxic cancer treatments. 17(8): 949 10.6004/jnccn.2019.7290 31390588

[14] Bright TJ, Wong A, Dhurjati R, Bristow E, Bastian L, Coeytaux RR, … Lobach D (2012) Effect of clinical decision-support systems: a systematic review*.* Ann Intern Med. 157(1): 29–43 10.7326/0003-4819-157-1-201207030-00450 22751758

[15] Panella M, Marchisio S, Di Stanislao F (2003) Reducing clinical variations with clinical pathways: do pathways work? Int J Qual Health Care. 15(6): 509–521 10.1093/intqhc/mzg057 14660534

[16] Zon RT, Frame JN, Neuss MN, Page RD, Wollins DS, Stranne S, and Bosserman LD (2016) American society of clinical oncology policy statement on clinical pathways in oncology. J Oncol Prac 12(3):261–266 10.1200/JOP.2015.009134 26759491

[17] Loprinzi CL, Lacchetti C, Bleeker J, Cavaletti G, Chauhan C, Hertz DL, … Hershman DL (2020) Prevention and management of chemotherapy-induced peripheral neuropathy in survivors of adult cancers: ASCO guideline update. J Clin Oncol. **0**(0): JCO.20.01399 10.1200/JCO.20.01399 32663120

[18] Jordan B, Margulies A, Cardoso F, Cavaletti G, Haugnes HS, Jahn P, … Jordan K (2020) Systemic anticancer therapy-induced peripheral and central neurotoxicity: ESMO-EONS-EANO clinical practice guidelines for diagnosis, prevention, treatment and follow-up. Ann Oncol. 31(10): 1306–1319 10.1016/j.annonc.2020.07.003 32739407

[19] Knoerl R, Mazzola E, Hong F, Salehi E, McCleary N, Ligibel J, … Berry DL (2021) Exploring the impact of a decision support algorithm to improve clinicians' chemotherapy-induced peripheral neuropathy assessment and management practices: a two-phase, longitudinal study. BMC Cancer 21(1): 236 10.1186/s12885-021-07965-8 PMC7937225 33676431

[20] Tanay MA, Armes J (2019) Lived experiences and support needs of women who developed chemotherapy-induced peripheral neuropathy following treatment for breast and ovarian cancer. Eur J Cancer Care 28(3):e13011 10.1111/ecc.13011 30790382

[21] Tanay MA, Armes J, Ream E (2017) The experience of chemotherapy-induced peripheral neuropathy in adult cancer patients: a qualitative thematic synthesis. Eur J Cancer Care 26(5):e12443 10.1111/ecc.12443 26786536

[22] Christopoulos D (2009) Peer esteem snowballing: a methodology for expert surveys. In: Eurostat conference for new techniques and technologies for statistics. Luxemburg 18–20

[23] Diamond IR, Grant RC, Feldman BM, Pencharz PB, Ling SC, Moore AM, Wales PW (2014). Defining consensus: a systematic review recommends methodologic criteria for reporting of Delphi studies. J Clin Epidemiol.

[24] Green B, Jones M, Hughes D, Williams A (1999). Applying the Delphi technique in a study of GPs' information requirements. Health Soc Care Community.

[25] Timmins HC, Mizrahi D, Li T, Kiernan MC, Goldstein D, Park SB (2021) Metabolic and lifestyle risk factors for chemotherapy-induced peripheral neuropathy in taxane and platinum-treated patients: a systematic review. J Cancer Surviv. 10.1007/s11764-021-00988-x 10.1007/s11764-021-00988-x 33438175

[26] Basch E, Deal AM, Kris MG, Scher HI, Hudis CA, Sabbatini P, … Schrag D (2016) Symptom monitoring with patient-reported outcomes during routine cancer treatment: a randomized controlled trial. J Clin Oncol. 34(6): 557–65 10.1200/JCO.2015.63.0830 PMC4872028 26644527

[27] Knoerl R, Smith EML, Han A, Doe A, Scott K, Berry DL (2019). Characterizing patient-clinician chemotherapy-induced peripheral neuropathy assessment and management communication approaches. Patient Educ Couns.

[28] McCrary JM, Goldstein D, Trinh T, Timmins HC, Li T, Friedlander M, … Park SB (2019) Optimizing clinical screening for chemotherapy-induced peripheral neuropathy. J Pain Symptom Manage. 58(6): 1023–1032 10.1016/j.jpainsymman.2019.07.021 31374367

[29] Yu A, Street D, Viney R, Goodall S, Pearce A, Haywood P, … Park SB (2021) Clinical assessment of chemotherapy-induced peripheral neuropathy: a discrete choice experiment of patient preferences. Support Care Cancer 29(11):6379–6387 10.1007/s00520-021-06196-8 33884508

[30] Vanhaecht K, De Witte K, Panella M, Sermeus W (2009). Do pathways lead to better organized care processes?. J Eval Clin Pract.

[31] Colquhoun HL, Squires JE, Kolehmainen N, Fraser C, Grimshaw JM (2017). Methods for designing interventions to change healthcare professionals’ behaviour: a systematic review. Implement Sci.

[32] Damschroder LJ, Aron DC, Keith RE, Kirsh SR, Alexander JA, Lowery JC (2009). Fostering implementation of health services research findings into practice: a consolidated framework for advancing implementation science. Implement Sci.

[33] Powell BJ, Waltz TJ, Chinman MJ, Damschroder LJ, Smith JL, Matthieu MM, … Kirchner JE (2015) A refined compilation of implementation strategies: results from the expert recommendations for implementing change (ERIC) project. Implement Sci. 10(1): 21 10.1186/s13012-015-0209-1 PMC4328074 25889199

[34] Salgado TM, Quinn CS, Krumbach EK, Wenceslao I, Gonzalez M, Reed HL, … Hertz DL (2020) Reporting of paclitaxel-induced peripheral neuropathy symptoms to clinicians among women with breast cancer: a qualitative study. Supportive Care in Cancer. 28(9): 4163–4172 10.1007/s00520-019-05254-6 PMC8261729 31897779

[35] Noonan VK, Lyddiatt A, Ware P, Jaglal SB, Riopelle RJ, Bingham CO, 3rd, … Ahmed S (2017) Montreal accord on patient-reported outcomes (PROs) use series - paper 3: patient-reported outcomes can facilitate shared decision-making and guide self-management. J Clin Epidemiol. 89: 125–135 10.1016/j.jclinepi.2017.04.017 28433671

[36] Hertz DL, Childs DS, Park SB, Faithfull S, Ke Y, Ali NT, … M. Lustberg (2021) Patient-centric decision framework for treatment alterations in patients with chemotherapy-induced peripheral neuropathy (CIPN). Cancer Treat Rev 99: 102241 10.1016/j.ctrv.2021.102241 34174668

[37] Kaku M, Vinik A, Simpson DM (2015). Pathways in the diagnosis and management of diabetic polyneuropathy. Curr Diab Rep.

[38] Mitchell AJ (2013). Screening for cancer-related distress: when is implementation successful and when is it unsuccessful?. Acta Oncol.

[39] Velasco R, Besora S, Argyriou AA, Santos C, Sala R, Izquierdo C, … Bruna J (2021) Duloxetine against symptomatic chemotherapy-induced peripheral neurotoxicity in cancer survivors: a real world, open-label experience. Anticancer Drugs. 32(1): 88–94 10.1097/CAD.0000000000001005 33332891

[40] Gewandter JS, Kleckner AS, Marshall JH, Brown JS, Curtis LH, Bautista J, … Mustian KM (2020) Chemotherapy-induced peripheral neuropathy (CIPN) and its treatment: an NIH collaboratory study of claims data. Support Care Cancer. 28(6): 2553–2562 10.1007/s00520-019-05063-x PMC7060096 31494735

[41] Cook C, Heath F, Thompson RL (2000) A meta-analysis of response rates in web- or internet-based surveys. Educat Psychol Meas 60(6):821–836

[42] Akins RB, Tolson H, Cole BR (2005). Stability of response characteristics of a Delphi panel: application of bootstrap data expansion. BMC Med Res Methodol.

