# Supplementary material for: Development and consensus process for a clinical pathway for the assessment and management of chemotherapy-induced peripheral neuropathy
Source: Support Care Cancer. 2022 Apr 8;30(7):5965–74. doi: 10.1007/s00520-022-07024-3 (PMC9135801; doi:10.1007/s00520-022-07024-3)
Supplement: Supplementary file 1 — Supplementary file1 (PDF 858 kb) [file 520_2022_7024_MOESM1_ESM.pdf]


Clinical Practice Pathway for the Assessment and Management of Chemotherapy-Induced Peripheral Neuropathy

---

Table of Contents

1. Background and clinical context....3

2. Pre-treatment....5

2.1. Pre-treatment assessment....5

2.2. Patient education and resources....6

3. During treatment....7

3.1. Assessment tools....7

3.2. CIPN management....9

4. Post-treatment: Management beyond treatment and survivorship....13

5. Clinical example of assessment and management of CIPN by treating team....14

6. Clinician resources....16

References....17

Appendix 1. Escalation plan for CIPN assessment....20

Appendix 2. CIPN assessments....21

The Clinical Practice Pathway for the Assessment and Management of Chemotherapy-Included Peripheral Neuropathy was developed by Susanna Park, David Goldstein, David Mizrahi, and Louisa Robinson on behalf of the In FOCUS research team at The University of New South Wales and The University of Sydney.

This research was supported by the Sydney Health Partners Implementation Science Grant, Cancer Institute NSW Program Grant (No. 14/TPG/1-05) and a National Health and Medical Research Council of Australia (NHMRC) Project Grant (No. 1080521).

1. Background and clinical context

Chemotherapy-induced peripheral neuropathy (CIPN) is a common and major dose limiting side effect of chemotherapies including vinca alkaloids, platinum compounds, taxanes, thalidomide, proteasome inhibitors, eribulin, and brentuximab vedotin (Table 1) [1]. Most commonly, CIPN is sensory predominant [2], characterised by tingling and numbness in the extremities, loss of sensation and pain, usually with symmetrical involvement in a glove and stocking distribution. The incidence of CIPN varies with chemotherapy agent, and is typically cumulative depending on duration of exposure and dose. These symptoms can lead to functional deficits with dexterity and fine motor skills, reduced balance or impaired gait, resulting in reduced quality of life and greater risk of falls [3]. Motor nerve dysfunction can also occur with some chemotherapy types, producing muscle cramps and weakness. Similarly, autonomic nerve dysfunction occurs with some neurotoxic chemotherapies, leading to orthostatic hypotension and bladder or bowel disturbance. Accordingly, CIPN can produce a significant impact on quality of life and ability to perform activities of daily living [4].

Class	Key Agents	Neuropathy
Taxane	Paclitaxel, Docetaxel, Nab-paclitaxel	**Acute:** Myalgias / arthralgias often develop within 1-4 days following paclitaxel infusion<br>**Chronic:** Tingling and numbness in the extremities. Deficits in fine motor skills and walking ability
Platinum compound	Oxaliplatin, Cisplatin, Carboplatin	**Acute:** Oxaliplatin - Distal and mouth tingling, allodynia, fasciculations and cramps. exacerbated by cold exposure<br>**Chronic:** Tingling and numbness, progressing to functional disability
Vinca alkaloid	Vincristine	**Chronic:** Distal sensorimotor neuropathy, with numbness, tingling, burning sensations, sharp pain, muscle cramps, distal muscle weakness, functional disability with fine motor tasks and walking
Immunomodulatory/ antiangiogenic agent	Thalidomide, Lenalidomide	**Chronic:** Painful paresthesia and numbness in the hands and feet and numbness; Muscle cramps and weakness
Proteasome inhibitor	Bortezomib, ixazomib, carfilzomib	**Chronic**: Tingling, numbness, sharp /burning pain in lower limbs
Eribulin	Eribulin	**Chronic**: Low grade sensorimotor (sensory predominant) neuropathy
Epothilone	Ixabepilone	**Chronic:** Development of sensory and motor neuropathy
Antibody-drug conjugates	Brentuximab vedotin, trastuzumab emantsine	**Chronic:** Sensory and occasionally painful neuropathy

Table 1. A summary of CIPN symptoms by common chemotherapy agents [1, 5-8].

Over-two thirds of patients treated with neurotoxic chemotherapy develop CIPN by the end of treatment and up to 40% experience long lasting effects well after completion of treatment [9], making it a potentially debilitating side-effect both throughout treatment and into survivorship. During treatment, CIPN is a prominent cause of dose reductions and premature cessation of treatment [10]. Resolution of symptoms may occur with cessation of treatment, but for some patients CIPN may have significant long-lasting effects on overall function and quality of life [11, 12]. There is currently no preventative or neuroprotective strategies and limited treatment options.

This clinical practice pathway was developed by clinicians, nurses, and researchers to aid the assessment and management of CIPN in adult cancer patients. Clinical decision making tools have been shown to improve health care process measures across diverse settings [13]. There are currently limited pathways outlining best practice for the assessment and management of CIPN [14, 15]. This clinical practice pathway provides a framework, which may differ across centres and by resource availability, to aid clinical decision making.

This clinical practice pathway complements existing guidelines focusing on prevention and treatment of CIPN, such as from the 2020 American Society of Clinical Oncology (ASCO) [16] and the European Society of Medical Oncology guidelines (ESMO) [17], by creating a framework on how to translate the assessment and management of CIPN into routine clinical practice. It was developed to:

- Establish routine processes for CIPN assessment and management
- Highlight patient groups at-risk of CIPN
- Provide an assessment guide for clinical personnel across all phases of treatment
- Support decision making processes for managing patients with CIPN
- Optimise long-term patient outcomes with early identification and intervention

2. Pre-Treatment

2.1. Pre-treatment assessment

Screening and assessment for CIPN starts with pre-treatment review, which should include screening for pre-existing peripheral neuropathy and assessment of potential risk factors in all patients prior to commencement of potentially neurotoxic treatment.

Screening for pre-existing neuropathy should include identifying diagnosed pre-existing peripheral neuropathy (e.g. diabetic, alcohol-associated or other neuropathies) as well as asking the patient regarding potential undiagnosed symptoms of neuropathy (e.g. tingling, numbness, neuropathic pain, muscle weakness). This can include the use of a patient-reported outcome CIPN screening questionnaire (see section 3.1) to identify pre-existing symptoms.

A medical history should be obtained to identify potential risk factors for CIPN. Below are some examples of possible or identified risk-factors to consider in the context of CIPN [18-22]. This list is not exhaustive, and some risk factors may still be preliminary and require validation across multiple studies. Potential risk factors include:

- Pre-existing peripheral neuropathy (e.g. neuropathy caused by diabetes, alcohol use, HIV or inherited neuropathies such as Charcot-Marie-Tooth disease)
- Diseases which place the patient at higher risk e.g. diabetes
- Neuropathic pain
- Prior treatment with other neurotoxic medications including previous chemotherapy
- High body mass index
- Vitamin B deficiency
- Older age
- Low pre-treatment haemoglobin levels

If patients present with pre-existing peripheral neuropathy or neuropathic symptoms, the following should be considered:

- Conduct a general medical and neurological assessment (see section 3.1) and/or consider referral to a neurologist for baseline nerve conduction studies.
- Closer monitoring for neurotoxicity throughout their treatment including using longer-patient reported outcome measures (see Section 3.1).

In addition, patients with identified substantial risk factors for CIPN development may benefit from baseline neurological assessment and closer monitoring throughout treatment. Patients who present with multiple CIPN risk factors, who are due to receive neurotoxic chemotherapy, may warrant referral to a neurologist to conduct baseline and follow up nerve conduction studies.

2.2. Patient education and resources

Patient education is an essential component of the recognition and management of CIPN. Prior to commencement of treatment the patient must be provided with information about CIPN and its symptoms. This education needs to continue throughout their treatment and updated accordingly in relation to their symptoms.

Patient education should be tailored to the individual especially regarding patients from culturally and linguistically diverse (CALD) backgrounds. Patient education should include the following:

- Chemotherapies which can cause CIPN
- Key CIPN symptoms
- How to monitor CIPN symptoms
- Importance of reporting signs and symptoms as soon as they are noticed
- Importance of reporting changes in functional capacity including impaired balance, changes in gait or increased anxiety around risk of falls
- Importance of being physically active throughout treatment for general health
- Risk management strategies regarding falls prevention and prevention of thermal injury

Available education guides about the signs and symptoms of CIPN include:

  - eviQ
  - Cancer Council
  - JAMA Oncology

3. <u>During treatment</u>

3.1. During treatment CIPN assessment and screening

During treatment, CIPN should be formally assessed and documented at every treatment cycle. CIPN should be initially assessed with a short patient-reported screening tool ± a clinician-reported tool (see Box 1). For patients who report **‘mild’** or worse persisting sensory and/or motor symptoms identified in the short screening assessment, a longer patient-reported CIPN instrument should be considered to provide further information about symptoms and their functional consequences, particularly identifying those reporting neuropathic pain (see Box 2 and Appendix 1).

There are multiple tools available to assess, grade and monitor CIPN severity before, during and after treatment. Brief patient-reported CIPN assessment tools include the NCI-PRO-CTCAE or Patient Neurotoxicity Questionnaire which use 2-3 questions to address the severity of CIPN and its overall impact on patient function. The responses from this can be used to grade the patient’s CIPN using a brief clinician-reported grading scale such as the National Cancer Institute Common Terminology Criteria for Adverse Events (NCI-CTCAE) sensory and motor neuropathy symptoms. With appropriate training, the NCI-CTCAE scale correlates well with more comprehensive measures of CIPN [23]. However, in routine practice, it demonstrates low inter-rater reliability and under-reports CIPN compared to patient report [24, 25]. Thus, it should be used in conjunction with a patient-reported outcome measure and not as a stand-alone tool. Full details of assessment tools can be accessed in Appendix 2.

Box 1 Brief CIPN assessment tools

Brief patient-reported outcome questionnaire:

NCI-PRO-CTCAE [26] or **Patient Neurotoxicity Questionnaire** (PNQ) [27].
These tools include 2-3 questions addressing CIPN severity and its overall impact on patient function. *Use the responses from these short patient-report screening tools to grade the patient’s CIPN using a clinician led tool.*

Brief clinician-reported grading scale:

NCI-CTCAE neuropathy sensory and neuropathy motor subscales
Grade peripheral motor and sensory neuropathy based on severity of symptoms (0-5), but should not be used as a stand-alone tool.

These tools have been extensively reviewed [28-30], but there remains a lack of consensus on the gold standard assessment tool [31]. Evidence is building that a combination of clinician and patient-reported assessments provides the most comprehensive information about CIPN [28, 32]. Clinician-reported tools have been shown to under-report the prevalence and severity of symptoms compared to patient reports [24]. However, reliance solely on patient reporting may also introduce bias. Further, because CIPN symptom report is inherently subjective, it can affected by multiple factors, including difference in patient perception of symptoms [33], patient-clinician communication style [34] and other subjective elements. In routine clinical practice, there is a preference for shorter instruments for improved feasibility [31] (See Box 1). Using these screening questionnaires can help to facilitate opening up a clinical discussion between patients and clinicians.

Box 2 Other CIPN assessment tools

Longer patient-reported CIPN assessments can include:

* **FACT/GOG-Ntx-13** [35]
* **EORTC-QLQ-CIPN20** [36], or the
* **EviQ CIPN screening tool** (yet to be validated).

These scales include 13-20 questions comprising symptom severity and more specific details of functional effects. Depending on system availability, these questionnaires could be completed by patients prior to their clinical review and results fed back to the treating team.

Pain questionnaires

Patients who report neuropathic pain symptoms using the EORTC-QLQ-CIPN20 or the EviQ CIPN screening tool should also complete a pain symptom rating scale such as the Pain Numeric Rating Scale (**PNRS**) [37].

The FACT/GOG-Ntx-13 questionnaire may not capture neuropathic pain symptoms and should be supplemented with an additional measure such as the PNRS to assess pain. It is important to identify patients with prominent neuropathic pain as there are specific treatment strategies which may help ameliorate symptoms (see Section 3.2.3).

Physical neuropathy assessment

A physical neurological assessment is commonly used to identify clinical signs of neuropathy such as reduced deep tendon reflexes, light touch, proprioception, pinprick and/or vibration sensation [17]. These signs are combined with patient report in the composite neurological assessment tool the **Total Neuropathy Score-clinical version** [38] (© Johns Hopkins University), which incorporates assessment of: sensory and motor symptoms, strength, deep tendon reflexes pin-prick and vibration sensitivity.

Nerve conduction studies are the neurophysiological gold standard for neuropathy and typically demonstrate sensory predominant axonal loss in CIPN [29]. While research studies have demonstrated some utility in monitoring CIPN progression with nerve conduction studies, results do not always correlate with patient symptom report.

3.2. CIPN Management

Management of CIPN has been comprehensively addressed in the ASCO [16] and ESMO [17] guidelines, encompassing systematic, expert-based review of available evidence from clinical trials of treatment and prevention approaches. This clinical practice pathway draws on these guidelines to provide suggestions on how to manage patients who present with CIPN symptoms, particularly if they are increasing in severity, duration, painful or distribution.

There is currently a lack of preventative, neuroprotective or proven treatment strategies for CIPN symptoms. Accordingly, it is critical to investigate symptoms and their functional impact as well as secondary symptoms that may be exacerbated by CIPN in order to provide optimal supportive care. Important elements to consider include neuropathic pain, balance impairment and falls risk, and sleep disturbances which may warrant referral to additional care providers. Patient education (see Section 2.2) about CIPN symptom management is also important, and should consider safety measures to reduce risk of falls and thermal injury as well as foot care.

CIPN is broadly symmetrical and slowly progressive, so the presence of atypical clinical presentations or rapidly escalating symptoms should lead to consideration and exclusion of other neurological or general etiologies.

3.2.1. Dose adjustments

The treating team should use their clinical judgment whether prolonged, worsening, increasing duration, painful or greater distribution of CIPN symptoms experienced by patient warrants a *dose adjustment,* including *delaying, dose reduction, substitutions, altering frequency or method of delivery (e.g. subcutaneous vs intravenous Bortezomib delivery), or ceasing chemotherapy* if functional impairment or intolerable CIPN occurs as per the ASCO guidelines [16]. Discuss with the patient their risk of worsening CIPN vs benefit of continuing treatment including the effect on patient's quality of life. Apart from dose adjustment, to date there are no preventative approaches available for CIPN as identified in the 2020 ASCO guidelines [16]. This highlights the need for early identification of CIPN and the accurate grading of symptom severity to guide decision making about the need for dose adjustment.

3.2.2. Referrals to the multi-disciplinary team

CIPN symptoms with functional impact (such as effects on balance, fine motor function or gait) may warrant referral to other specialities. Potential referrals include:

Neurology

For assessment or management of symptoms, nerve conduction studies or specialist clinical review can be conducted by the neurology team to monitor nerve function over time to assess complications from treatment. If concerned about prolonged, worsening, increasing duration, painful or greater distribution of CIPN symptoms, consider referral to neurology services. It is important that rapid escalation of symptoms or sudden acute symptoms are considered for investigation by referral for clinical review/nerve conduction studies by a Neurologist. Nerve conduction studies can provide additional information about nerve pathology and rule out other conditions.

Allied health services

Functional impairments as identified using a longer patient-reported CIPN questionnaire may warrant referral to multi-disciplinary allied health services. Appropriate referral to allied health can enhance patient care through their provision of practical assistance, advice and patient education.

Exercise Physiologist/Physiotherapist

Patients displaying changes in gait, balance concerns or increased risk of falls should be referred to an exercise physiologist or physiotherapist for assessment and guidance. There is some evidence that balance training and exercise demonstrates benefits in patients with CIPN to improve functional capacity and quality life [39, 40]. Resistance exercises can strengthen muscles weakened by neuropathy. Balance exercise can assist in improving impaired balance from CIPN. This is important both in the active phase of treatment and survivorship. The Clinical Oncology Society of Australia (COSA) position statement on exercise in cancer care recommends referral to an exercise physiologist or physiotherapist with experience in cancer care to provide a tailored exercise program taking into consideration individualized needs and functional impairments [41].

Occupational Therapist

An occupational therapist can assist patients who experience a level of disability and impaired activities of living as a result of CIPN. They can educate the patient on risk

management regarding falls or change in sensation. They can assess the patient's functional assessment and provide strategies to manage these such as aids to assist with activities of daily living.

Other services

In addition to the above allied health professionals, based on the needs of each patient, the treating team could consider the role of a psychologist, social worker and/or podiatrist in helping to manage impacts to patient's lives due to CIPN symptoms. If CIPN produces prominent pain, this may warrant referral to a pain service. Similarly, referral may be warranted for management of sleep disturbances.

3.2.3. Treatment strategies

Pharmacological agents

Consider pharmacological intervention to treat CIPN. As per current guidelines [16, 17]:

- **Symptomatic treatment:** Consider prescribing Duloxetine for *painful* CIPN (evidence among patients treated with Taxanes or Oxaliplatin) [42]. Duloxetine is the only agent that level 1 evidence to support its use for patients with established painful CIPN, although the amount of benefit is limited [16, 17].
- **Symptomatic treatment:** There is less available evidence for other pharmacological agents for treatment of neuropathic pain in the context of CIPN but inconclusive evidence is available for venlafaxine, pregabalin, amitriptyline, tramadol and opioids [17].
- Inconclusive scientific evidence exists [16] for the following agents treat CIPN, which could be considered in selected patients with CIPN after discussing the limited scientific evidence, potential harms, benefits, costs and patient preferences:
    - Tricyclic antidepressants
    - Gabapentin/pregabalin
    - Oral cannabinoids
    - A topical gel treatment containing baclofen (10 mg), amitriptyline HCL (40 mg), and ketamine (20 mg)
    - Topical low concentration menthol

Supportive care

Although the 2020 ASCO guidelines have not recommended the following non-pharmacological interventions to treat CIPN, they have suggested there is preliminary evidence of potential benefit, but will require validation in larger studies prior to recommendation [16]:

- Physical activity
- Acupuncture
- Scrambler therapy

4. Post-treatment: Management beyond treatment and survivorship

For some patients, the symptoms of CIPN may be transient and reverse upon cessation or completion of treatment, but up to 40% of patients have long lasting symptoms that persist long after treatment, and may be permanent [1]. Additionally, a "coasting effect" can also occur and patients may develop CIPN symptoms 3-6 months after ceasing treatment [43].

Thus, it is essential that the patient continues to be followed up after completion of treatment. For patients with persisting or coasting CIPN, or with identified functional deficits, an identical pathway approach should be considered including:

- Assess for signs of improvement or deterioration using questionnaires and questions about activities of daily living (such as EORTC QLQ-CIPN20, FACT/GOG-Ntx-13) (see Section 3.1).
- Consider conducting similar physical neuropathy assessments (see Section 3.1)
- Consider referral to allied health such as exercise physiologist/physiotherapist or occupational therapist with the goal to improve functional capacity and performance of activities of daily living if these are affected (see Section 3.2.2).
    - These services can also be considered for patients who are at increased risk of falls or increased anxiety about balance who could potentially benefit from a structured exercise program.
- Consider a referral to an expert in late treatment-related toxicities such as a neurologist, pain specialist, or management for sleep disturbances.
- Consider prescribing Duloxetine for painful CIPN (As per the 2020 ASCO guidelines; see Section 3.2.3).

5. Clinical example of assessment and management

Below are examples of action points and escalation of care regarding CIPN symptoms, functional impairment, and pain:

Mild CIPN symptoms (using short patient-reported questionnaire)

- Be alert/aware of the change in the patient's symptoms and use a longer patient-reported CIPN and pain questionnaire to identify if there are any changes in physical function or pain.
- Discuss with the patient their risk of worsening CIPN vs benefit of continuing the same treatment intensity including the effect on patient's quality of life.
- Provide patient education on managing symptoms and when to report signs/symptoms to the healthcare team.
- Consider adding an ongoing alert/prompt to the electronic medical records or a note on the patients file flagging that the patient has self-reported CIPN symptoms.

If you or the patient are concerned about worsening, prolonged, greater distribution or longer CIPN symptoms:

- **Referral:** Consider referral to Neurologist for detailed neurological assessment
  Referral: Consider referral to GP for assessment and potential management of other symptoms as a result of CIPN (e.g. sleep disturbance)
- **Dose adjustment:** Treating oncologist to use clinical judgment whether they consider the appropriateness of *dose delaying, dose reduction, substitutions or ceasing chemotherapy* if functional impairment or intolerable CIPN occurs as per the ASCO guidelines [16]. Discuss with patient their risk of worsening CIPN vs benefit of continuing treatment including the effect on patient's quality of life.

If the patient reports painful symptoms

- **Referral**: Consider referral to Neurologist for detailed neurological assessment
- **Referral**: Consider referral to pain specialist
- **Prescription:** Consider referring to 2020 ASCO guidelines for pharmacological intervention including Duloxetine for *painful* CIPN [16]
- **Dose adjustment:** Treating oncologist to use clinical judgment whether they consider the appropriateness of *dose delaying, dose reduction, substitutions or ceasing chemotherapy* if functional impairment or intolerable CIPN occurs as per the ASCO guidelines [16]. Discuss with patient their risk of worsening CIPN vs benefit of continuing treatment including the effect on patient's quality of life.

If the patient reports functional impairments including reduced balance or anxiety of falling (using long patient-reported form)

- **Referral:** Consider referral to allied health services (such as exercise physiology, physiotherapy or occupational therapy)
- **Dose adjustment:** Treating oncologist to use clinical judgment whether they consider the appropriateness of *dose delaying, dose reduction, substitutions or ceasing chemotherapy* if functional impairment or intolerable CIPN occurs as per the ASCO guidelines [16]. Discuss with patient their risk of worsening CIPN vs benefit of continuing treatment including the effect on patient's quality of life.

A clinical practice pathway for chemotherapy-induced peripheral neuropathy assessment and management

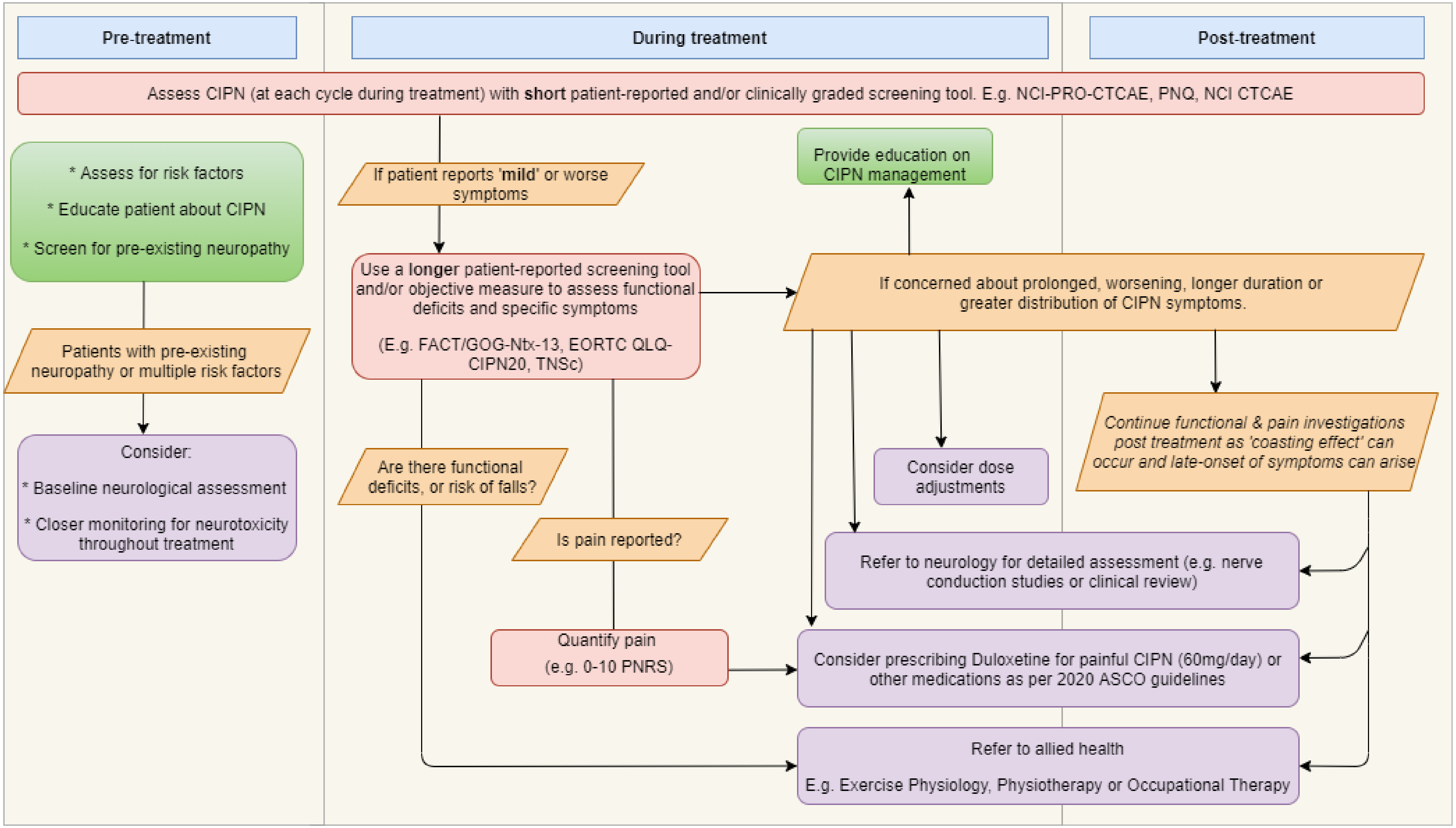

6. Clinical resources

- 2020 ASCO guidelines
- 2020 ESMO guidelines
- CIPN assessment tools
    - NCI-CTCAE (clinician graded short scale)
    - NCI-PRO-CTCAE (patient-reported short questionnaire)
    - EORTC QLQ-CIPN20 (patient-reported detailed questionnaire)
    - FACT/GOG-Ntx-13 (patient-reported detailed questionnaire)
    - eviQ (patient-reported detailed questionnaire - *yet to be validated*)

References

1. Park, S.B., et al., *Chemotherapy-induced peripheral neurotoxicity: A critical analysis.* CA: A Cancer Journal for Clinicians, 2013. **63**(6): p. 419-437.
2. Seretny, M., et al., *Incidence, prevalence, and predictors of chemotherapy-induced peripheral neuropathy: A systematic review and meta-analysis.* Pain, 2014. **155**(12): p. 2461-2470.
3. Winters-Stone, K.M., et al., *Falls, Functioning, and Disability Among Women With Persistent Symptoms of Chemotherapy-Induced Peripheral Neuropathy.* Journal of clinical oncology : official journal of the American Society of Clinical Oncology, 2017. **35**(23): p. 2604-2612.
4. Mols, F., et al., *Chemotherapy-induced peripheral neuropathy and its association with quality of life: a systematic review.* Support Care Cancer, 2014. **22**(8): p. 2261-9.
5. Timmins, H.C., et al., *Quantification of Small Fiber Neuropathy in Chemotherapy-Treated Patients.* J Pain, 2019.
6. Li, T., et al., *Peripheral neuropathy in hematologic malignancies – Past, present and future.* Blood Reviews, 2020: p. 100653.
7. Tamburin, S., et al., *Taxane and epothilone-induced peripheral neurotoxicity: From pathogenesis to treatment.* J Peripher Nerv Syst, 2019. **24 Suppl 2**: p. S40-s51.
8. Islam, B., et al., *Vinca alkaloids, thalidomide and eribulin-induced peripheral neurotoxicity: From pathogenesis to treatment.* J Peripher Nerv Syst, 2019. **24 Suppl 2**: p. S63-s73.
9. Wolf, S., et al., *Chemotherapy-induced peripheral neuropathy: prevention and treatment strategies.* Eur J Cancer, 2008. **44**(11): p. 1507-15.
10. Mileshkin, L., et al., *Development of neuropathy in patients with myeloma treated with thalidomide: patterns of occurrence and the role of electrophysiologic monitoring.* J Clin Oncol, 2006. **24**(27): p. 4507-14.
11. Richardson, P.G., et al., *Reversibility of symptomatic peripheral neuropathy with bortezomib in the phase III APEX trial in relapsed multiple myeloma: impact of a dose-modification guideline.* Br J Haematol, 2009. **144**(6): p. 895-903.
12. Battaglini, E., et al., *Chemotherapy-induced peripheral neurotoxicity in cancer survivors: predictors of long-term patient outcomes.* Journal of the National Comprehensive Cancer Network, 2021.
13. Bright, T.J., et al., *Effect of clinical decision-support systems: a systematic review.* Ann Intern Med, 2012. **157**(1): p. 29-43.
14. Knoerl, R., et al., *Exploring the efficacy of an electronic symptom assessment and self-care intervention to preserve physical function in individuals receiving neurotoxic chemotherapy.* BMC Cancer, 2018. **18**(1): p. 1203.
15. Tofthagen, C., C.M. Visovsky, and R. Hopgood, *Chemotherapy-induced peripheral neuropathy: an algorithm to guide nursing management.* Clin J Oncol Nurs, 2013. **17**(2): p. 138-44.
16. Loprinzi, C.L., et al., *Prevention and Management of Chemotherapy-Induced Peripheral Neuropathy in Survivors of Adult Cancers: ASCO Guideline Update.* Journal of Clinical Oncology, 2020. **0**(0): p. JCO.20.01399.
17. Jordan, B., et al., *Systemic anticancer therapy-induced peripheral and central neurotoxicity: ESMO-EONS-EANO Clinical Practice Guidelines for diagnosis, prevention, treatment and follow-up.* Annals of Oncology, 2020. **31**(10): p. 1306-1319.
18. Mizrahi, D., et al., *Hemoglobin, Body Mass Index, and Age as Risk Factors for Paclitaxel- and Oxaliplatin-Induced Peripheral Neuropathy.* JAMA Network Open, 2021. **4**(2): p. e2036695-e2036695.
19. Greenlee, H., et al., *BMI, Lifestyle Factors and Taxane-Induced Neuropathy in Breast Cancer Patients: The Pathways Study.* JNCI: Journal of the National Cancer Institute, 2016. **109**(2).
20. Hershman, D.L., et al., *Comorbidities and Risk of Chemotherapy-Induced Peripheral Neuropathy Among Participants 65 Years or Older in Southwest Oncology Group Clinical Trials.* J Clin Oncol, 2016. **34**(25): p. 3014-22.

21. Timmins, H.C., et al., *Metabolic and lifestyle risk factors for chemotherapy-induced peripheral neuropathy in taxane and platinum-treated patients: a systematic review.* Journal of Cancer Survivorship, 2021.
22. Molassiotis, A., et al., *Risk factors for chemotherapy-induced peripheral neuropathy in patients receiving taxane- and platinum-based chemotherapy.* Brain Behav, 2019. **9**(6): p. e01312.
23. Cavaletti, G., et al., *The Total Neuropathy Score as an assessment tool for grading the course of chemotherapy-induced peripheral neurotoxicity: comparison with the National Cancer Institute-Common Toxicity Scale.* J Peripher Nerv Syst, 2007. **12**(3): p. 210-5.
24. Tan, A.C., et al., *Chemotherapy-induced peripheral neuropathy-patient-reported outcomes compared with NCI-CTCAE grade.* Support Care Cancer, 2019. **27**(12): p. 4771-4777.
25. Molassiotis, A., et al., *Are we mis-estimating chemotherapy-induced peripheral neuropathy? Analysis of assessment methodologies from a prospective, multinational, longitudinal cohort study of patients receiving neurotoxic chemotherapy.* BMC Cancer, 2019. **19**(1): p. 132.
26. Dueck, A.C., et al., *Validity and Reliability of the US National Cancer Institute's Patient-Reported Outcomes Version of the Common Terminology Criteria for Adverse Events (PRO-CTCAE).* JAMA Oncol, 2015. **1**(8): p. 1051-9.
27. Shimozuma, K., et al., *Feasibility and validity of the Patient Neurotoxicity Questionnaire during taxane chemotherapy in a phase III randomized trial in patients with breast cancer: N-SAS BC 02.* Supportive Care in Cancer, 2009. **17**(12): p. 1483.
28. Park, S.B., et al., *Overview and critical revision of clinical assessment tools in chemotherapy-induced peripheral neurotoxicity.* Journal of the Peripheral Nervous System, 2019. **24**(S2): p. S13-S25.
29. Argyriou, A.A., et al., *Neurophysiological, nerve imaging and other techniques to assess chemotherapy-induced peripheral neurotoxicity in the clinical and research settings.* 2019. **90**(12): p. 1361-1369.
30. Cavaletti, G., et al., *Chemotherapy-Induced Peripheral Neurotoxicity assessment: a critical revision of the currently available tools.* Eur J Cancer, 2010. **46**(3): p. 479-94.
31. McCrary, J.M., et al., *Optimal clinical assessment strategies for chemotherapy-induced peripheral neuropathy (CIPN): a systematic review and Delphi survey.* Support Care Cancer, 2017. **25**(11): p. 3485-3493.
32. Alberti, P., et al., *Physician-assessed and patient-reported outcome measures in chemotherapy-induced sensory peripheral neurotoxicity: two sides of the same coin.* Ann Oncol, 2014. **25**(1): p. 257-64.
33. Salgado, T.M., et al., *Patient factors associated with discrepancies between patient-reported and clinician-documented peripheral neuropathy in women with breast cancer receiving paclitaxel: A pilot study.* 2020. **51**: p. 21-28.
34. Knoerl, R., et al., *Characterizing patient-clinician chemotherapy-induced peripheral neuropathy assessment and management communication approaches.* Patient Education and Counseling, 2019. **102**(9): p. 1636-1643.
35. Atkinson, T.M., et al., *The association between clinician-based common terminology criteria for adverse events (CTCAE) and patient-reported outcomes (PRO): a systematic review.* Support Care Cancer, 2016. **24**(8): p. 3669-76.
36. Le-Rademacher, J., et al., *Patient-reported (EORTC QLQ-CIPN20) versus physician-reported (CTCAE) quantification of oxaliplatin- and paclitaxel/carboplatin-induced peripheral neuropathy in NCCTG/Alliance clinical trials.* Supportive Care in Cancer, 2017. **25**(11): p. 3537-3544.
37. Farrar, J.T., et al., *Clinical importance of changes in chronic pain intensity measured on an 11-point numerical pain rating scale.* PAIN, 2001. **94**(2): p. 149-158.
38. Cornblath, D.R., et al., *Total neuropathy score: validation and reliability study.* Neurology, 1999. **53**(8): p. 1660-4.

39. McCrary, J.M., et al., *Exercise-based rehabilitation for cancer survivors with chemotherapy-induced peripheral neuropathy.* Support Care Cancer, 2019. **27**(10): p. 3849-3857.
40. Kanzawa-Lee, G.A., et al., *Exercise Effects on Chemotherapy-Induced Peripheral Neuropathy: A Comprehensive Integrative Review.* Cancer Nurs, 2020. **43**(3): p. E172-e185.
41. Cormie, P., et al., *Clinical Oncology Society of Australia position statement on exercise in cancer care.* Med J Aust, 2018.
42. Smith, E.M., et al., *Effect of duloxetine on pain, function, and quality of life among patients with chemotherapy-induced painful peripheral neuropathy: a randomized clinical trial.* Jama, 2013. **309**(13): p. 1359-67.
43. Gupta, R. and A. Bhaskar, *Chemotherapy-induced peripheral neuropathic pain.* BJA Education, 2015. **16**(4): p. 115-119.

Website links to clinical resources

1. 2020 ASCO guidelines - https://ascopubs.org/doi/pdf/10.1200/JCO.20.01399
2. 2020 ESMO guidelines - https://www.annalsofoncology.org/article/S0923-7534(20)39938-5/abstract
3. NCI-CTCAE clinician graded scale - https://evs.nci.nih.gov/ftp1/CTCAE/CTCAE_4.03/CTCAE_4.03_2010-06-14_QuickReference_8.5x11.pdf
4. NCI-PRO-CTCAE questionnaire - https://healthcaredelivery.cancer.gov/pro-ctcae/pro-ctcae_english.pdf
5. EORTC QLQ-CIPN20 questionnaire - https://qol.eortc.org/questionnaire/qlq-cipn20/
6. FACT/GOG-Ntx-13 questionnaire - https://www.facit.org/measures/FACT-GOG-NTX-13
7. EviQ questionnaire - https://www.eviq.org.au/clinical-resources/assessment-tools/8-chemotherapy-induced-peripheral-neuropathy-scree

Website links for patient resources

1. EviQ - https://www.eviq.org.au/patients-and-carers/patient-information-sheets/managing-side-effects/536-peripheral-neuropathy-during-cancer-treatment
2. Cancer Council - https://www.cancer.org.au/assets/pdf/understanding-peripheral-neuropathy#:~:text=What%20causes%20this%20nerve%20damage,itself%20can%20cause%20peripheral%20neuropathy.
3. JAMA Oncology - https://jamanetwork.com/journals/jamaoncology/fullarticle/2726030

Appendix 1. Assessment escalation plan

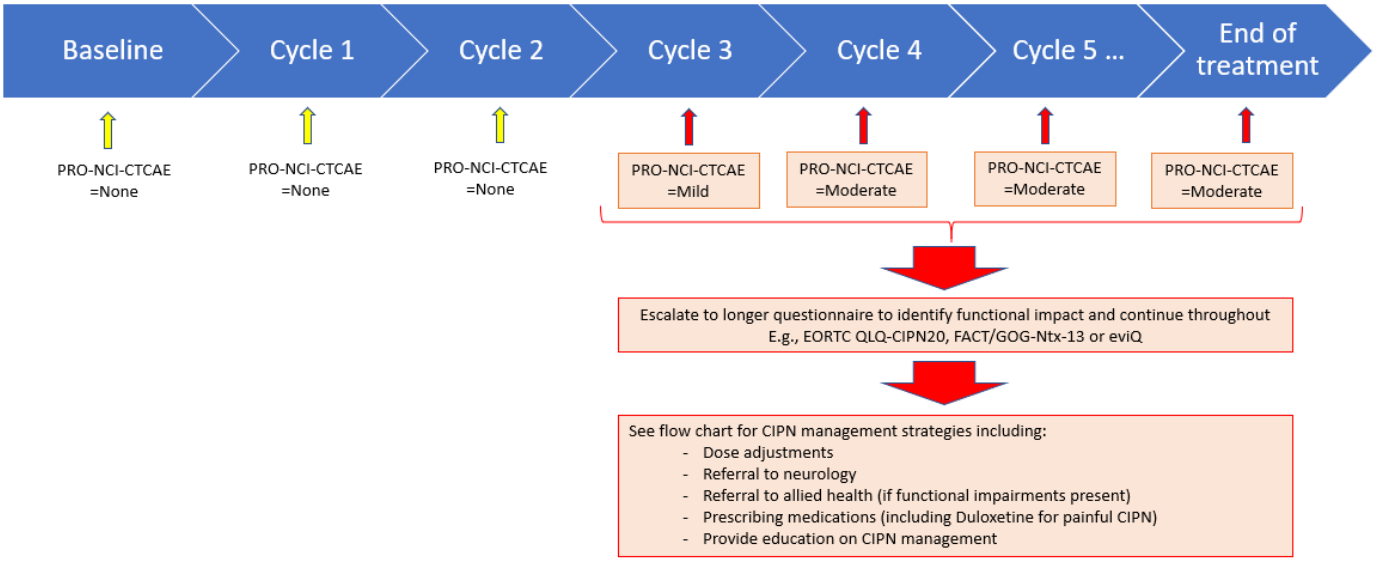

Figure 2. An example of escalating CIPN assessment from using the short tool only (with no/mild CIPN symptoms) to including the addition of a longer tool to identify any functional impacts with the presence of moderate or severe CIPN symptoms.

Appendix 2. CIPN assessments

Short screening assessments

National Cancer Institute Patient Reported Outcomes Common Terminology Criteria for Adverse Events (NCI-PRO-CTCAE)

The NCI-PRO-CTCAE (question 39) involves asking the patient 2 questions about the severity of numbness and tingling and how much this interfered with their daily activities. It is graded from 'none' to 'very severe/much' [26].

In the last 7 days, what was the SEVERITY of your NUMBNESS OR TINGLING IN YOUR HANDS OR FEET at its WORST?

None	Mild	Moderate	Severe	Very severe
☐	☐	☐	☐	☐

In the last 7 days, how much did NUMBNESS OR TINGLING IN YOUR HANDS OR FEET INTERFERE with your usual or daily activities?

Not at all	A little bit	Somewhat	Quite a bit	Very much
☐	☐	☐	☐	☐

Figure 3. NCI-PRO-CTCAE patient-reported questionnaire

Patient Neurotoxicity Questionnaire (PNQ)

The PNQ is a 2-item patient reported screening tool. It asks the patient to grade their numbness, pain, tingling, breathing, swallowing and muscle spasms from 0-4. It also provides examples of activities of daily living that may be affected. The PNQ has a strong correlation with the longer patient-reported questionnaire FACT/GOG-Ntx-13 [27].

Figure 4. Patient Neurotoxicity Questionnaire patient-reported questionnaire

Item 1

☐ A	☐ B	☐ C	☐ D	☐ E
I have no numbness, pain, burning, tingling or change in my sense of touch in my hands/fingers, or feet/toes or mouth area.	I have mild numbness, burning, pain, tingling or change in my sense of touch in my hands/fingers, or feet/ toes or mouth area. This does not interfere with my activities of daily living.	I have moderate burning, numbness, pain, tingling or change in my sense of touch in my hands/fingers, or feet/toes or mouth area. This does not interfere with my activities of daily living.	I have moderate to severe burning, numbness, pain, tingling or change in my sense of touch in my hands/fingers, or feet/toes or mouth area. This interferes with my activities of daily living.	I have severe numbness, pain, tingling or change in my sense of touch in my hands/fingers, or feet/toes or mouth area. It completely prevents me from doing most activities of daily living.

Item 2

☐ A	☐ B	☐ C	☐ D	☐ E
I have no difficulty in swallowing, breathing, drinking or chewing food, or muscle spasms in my mouth/jaws, hands/fingers, or feet/toes.	I have a mild difficulty in swallowing, breathing, drinking or chewing food, or muscle spasms, in my mouth/ jaws, hands/fingers or feet/ toes, This does not interfere with my activities of daily living.	I have moderate difficulty in swallowing, breathing, drinking or chewing food, or muscle spasms, in my mouth/jaws, hands/fingers, or feet/toes. This does not interfere with my activities of daily living.	I have moderate to severe difficulty in swallowing, breathing, drinking or chewing food, or muscle spasms in my mouth/jaws, hands/fingers, or feet/toes. This interferes with my activities of daily living.	I have severe difficulty in swallowing, breathing, drinking or chewing food, or muscle spasms in my mouth/jaws, hands/fingers, or feet/toes. It completely prevents me from doing most activities of daily living.

• Please indicate by placing an x in the box or writing in the space provided which activity or activities have been interfered with as a result of therapy.

My ability to:

☐ Button clothes	☐ Zippers	☐ Fasten buckles	☐ Write	☐ Sew
☐ Use a knife	☐ Put in or remove contact lenses	☐ Sleep	☐ Walk	☐ Work
☐ Use a fork		☐ Climb stairs	☐ Put on jewelry	☐ Tie shoes
☐ Use a spoon	☐ Dial or use telephone	☐ Type on a keyboard	☐ Knit	☐ Drive
☐ Swallowing	☐ Operate a remote control	☐ Eating/chewing	☐ Drinking liquids	☐ Shortness of breath
☐ Open doors	☐ Use other eating utensils	☐ Work or perform activities of importance to me, specify:		

National Cancer Institute Common Terminology Criteria for Adverse Events (NCI CTCAE)

The NCI CTCAE is a brief clinician-reported grading scale and is very commonly used clinically. The NCI CTCAE contains single-item grading scales, and can be used to grade both peripheral sensory neuropathy and peripheral motor neuropathy based on severity of symptoms from no symptoms (grade 0) to symptoms of increasing severity and impact on daily function (grades 1-5). With appropriate training, the NCI CTCAE scale correlates well with more comprehensive measures of CIPN [23].

**Nervous system disorders**					
	**Grade**				
**Adverse Event**	**1**	**2**	**3**	**4**	**5**
Peripheral motor neuropathy	Asymptomatic; clinical or diagnostic observations only; intervention not indicated	Moderate symptoms; limiting instrumental ADL	Severe symptoms; limiting self care ADL; assistive device indicated	Life-threatening consequences; urgent intervention indicated	Death
Definition: A disorder characterized by inflammation or degeneration of the peripheral motor nerves.					
Peripheral sensory neuropathy	Asymptomatic; loss of deep tendon reflexes or paresthesia	Moderate symptoms; limiting instrumental ADL	Severe symptoms; limiting self care ADL	Life-threatening consequences; urgent intervention indicated	Death
Definition: A disorder characterized by inflammation or degeneration of the peripheral sensory nerves.					

Figure 5. National Cancer Institute Common Terminology Criteria for Adverse Events (NCI CTCAE) for peripheral motor neuropathy and peripheral sensory neuropathy to be graded by a nurse or oncologist.

Longer patient-reported assessments

Functional Assessment of Cancer Therapy/Gynaecologic Oncology Group Neurotoxicity Tool (FACT/GOG-Ntx-13)

The FACT/GOG-Ntx-13 Questionnaire is a 13-item patient reported assessment. The patient completes questions which ask about CIPN symptoms and rate how much they are experiencing these from not at all (0) to very much (4). The FACT/GOG-Ntx-13 has displayed a moderate correlation with the CTCAE [35].

European Organisation for Research and Treatment of Cancer Quality of Life Questionnaire (EORTC QLQ-CIPN20)

The EORTC QLQ-CIPN20 is a 20-item patient-reported assessment. The patient is asked about their CIPN symptoms either describing certain symptoms such as tingling or numbness and relating to activities of daily living such as difficulty holding a pen or opening a jar. They then rate not at all (1) to very much (4). As with other treatment-related side effects, there can be a discrepancy between clinician rated and patient rated severity. The EORTC QLQ-CIPN20 has been shown to have a strong correlation with NCI-CTCAE, although there is no reliable scoring conversion available for the individual patient [36].

EviQ Chemotherapy induced peripheral neuropathy (CIPN) screening tool

The EviQ CIPN screening tool is an 11-question questionnaire in which the clinician grades the patient's symptoms from none (0) to disabling/life threatening (4). It uses sensory descriptors as well as task related questions and includes 2 question about autonomic neuropathy (constipation and swallow). ***This questionnaire has yet to be validated or compared against other validated measures***.

Total Neuropathy Score clinical version (TNSc)

The TNSc is a clinical composite grading scale to assess neurological function, incorporating both sensory and motor assessment [17, 38]. Each domain is graded between 0 (normal) and 4 (severe), with a global score calculated by adding scores from all domains.

These include assessment and grading of:

- Patient reported sensory and motor neuropathy symptoms
- Upper and lower limb pin-prick sensibility
- Upper and lower limb vibration sensibility
- Deep tendon reflexes
- Muscle strength assessment

Item	0	1	2	3	4
Sensory symptoms (numbness, tingling, and neuropathic pain)	None	Limited to fingers or toes	Extension to ankle or wrist	Extension to knee or elbow	Above knees or elbows or functionally disabling
Motor symptoms	None	Slight difficulty	Moderate difficulty	Assistance required	Paralysis
Pin sensibility	Normal	Reduced in fingers or toes	Reduced to wrist or ankle	Reduced to elbow or knee	Reduced above elbow or knee
Vibration sensibility	Normal	Reduced in fingers or toes	Reduced to wrist or ankle	Reduced to elbow or knee	Reduced above elbow or knee
Strength	Normal	Mild weakness	Moderate weakness	Severe weakness	Paralysis
Tendon reflexes	Normal	Ankle reflex reduced	Ankle reflex absent	Ankle reflex absent or others reduced	All reflexes absent
